# Level of Knowledge on Stroke and Associated Factors: A Cross-Sectional Study at Primary Health Care Centers in Morocco

**DOI:** 10.5334/aogh.2885

**Published:** 2020-07-23

**Authors:** Ahmed Kharbach, Majdouline Obtel, Abderrahmane Achbani, Youssef Bouchriti, Kenza Hassouni, Laila Lahlou, Rachid Razine

**Affiliations:** 1 Laboratory of Biostatistics, Clinical Research and Epidemiology (LBRCE), Faculty of Medicine and Pharmacy of Rabat, Mohammed V University of Rabat, MA; 2 Laboratory of Social Medicine (Public Health, Hygiene and Preventive Medicine), Faculty of Medicine and Pharmacy of Rabat, Mohammed V University of Rabat, MA; 3 Laboratory of Cell Biology and Molecular Genetics (LBCGM), Department of Biology, Faculty of Sciences, University Ibn Zohr, Agadir, MA; 4 Faculty of Sciences, University Ibn Zohr, Agadir, MA; 5 International School of Public Health, Mohammed VI University of Health Sciences (UM6SS), Casablanca, MA; 6 Faculty of Medicine and Pharmacy of Agadir, University Ibn Zohr, Agadir, MA

## Abstract

**Background:**

Stroke is increasingly becoming a major cause of disability and mortality. However, it can be prevented by raising awareness about risk factors and early health care management of patients.

**Objective:**

The aim of this study is to assess the level of knowledge on stroke, its risk factors, and warning signs in the population attending urban primary health care centers in the city of Agadir, Morocco.

**Methods:**

This is a multicentric cross-sectional study with a descriptive and analytical purpose. The study was conducted at five urban primary health care centers in Agadir in centralwest Morocco. All persons over the age of 18 years who consulted the health centers and who agreed to fill in the questionnaire were recruited, except for the foreign population and health workers. An interview questionnaire was used to assess the level of knowledge on stroke.

**Findings:**

A total of 469 participants were involved in the study. The median knowledge score was 8 (Interquartile range 4–13). High blood pressure (55.7%), depression and stress (48.8%) were the most well-known risk factors. Sudden weakness of the face, arms or legs (37.3%) was the main warning sign cited by the participants. Multivariate analysis revealed that illiteracy (OR 1.92; CI_95%_: 1.08–3.44) primary education (OR 3.43; CI_95%_: 1.63–7.21), rural residential (OR 1.67; CI_95%_: 1.07–2.59), no history of stroke among respondents (OR 16.41; CI_95%_: 4.37–61.59) and no history of stroke among relatives, acquaintances, or neighbors (OR 4.42; CI_95%_: 2.81–6.96), were independently associated with a lower level of knowledge of stroke (Table 4).

**Conclusions:**

The low level of knowledge on stroke among this Moroccan population indicates the importance of implementing stroke education initiatives in the community. More specifically, proximity education and awareness programs ought to be considered to anchor lifestyle preventive behaviors along with appropriate and urgent actions regarding the warning signs of stroke.

## Introduction

Stroke is the second leading cause of mortality and disability worldwide, with more than 13 million new cases per year, and is associated with an increased economic burden due to different treatments and post-stroke care [1,2].

Stroke incidence and mortality have increased in the countries of the Middle East and North Africa over the past decade, and projections indicate that stroke-related deaths will approximately double by 2030 in the same region [3,4]. Despite the rapid evolution of the generalization of intravenous thrombolysis in recent years in the countries of the Middle East and North Africa [5], the average symptom onset to arrival at a stroke center or emergency department (onset-to-door time [ODT]) of patients with ischemic stroke in Morocco remains very long, possibly resulting from a lack of knowledge, particularly of the first warning signs of an ischemic stroke, according to a recent systematic review [6].

Consequently, insufficient knowledge of risk factors, warning signs, and urgent therapeutic approach options have been identified as a serious cause of increased mortality and morbidity due to stroke [7]. Similarly, this knowledge deficiency has been identified as one of the significant barriers to accessing quality health care for stroke in Africa, as well as a factor affecting pre-hospital time [8,9]. Several studies in various countries have all confirmed the persistence of a low level of knowledge among the general public about stroke, and more specifically about risk factors and warning signs [10,11,12,13].

In Morocco, no previous study has been published exploring the level of knowledge of the Moroccan population about stroke. For this reason, the present investigation represents a first proposal in Morocco to assess the level of knowledge about stroke, as well as the factors associated with it, among people attending health centers belonging to the network of primary health care centers in Agadir in central-western Morocco.

## Methods

### Design and study area

This study involved a cross-sectional survey with a descriptive and analytical aim, conducted in five urban primary health care centers in Agadir, in the Souss Massa region in the center-west of Morocco. Agadir Ida-Outanane province is located in central-western Morocco. It covers an area of 2297 km^2^, with a total population of 600,599 inhabitants [14].

### Inclusion and Exclusion Criteria

Participants, aged 18 and over (patients, patients’ companions and visitors), attending urban primary health care centers held as part of the study to benefit from preventive or curative care, were included in the study. The foreign population (non-Moroccan) and health workers were excluded.

### Sample and recruitment of study participants

The sample size was calculated based on a 5.0% error range, a 95% confidence interval (CI) for a total Moroccan population of 600,599 inhabitants in the province of Agadir Ida-Outanane [14], and an anticipated population proportion of stroke knowledge deficiency of 50%. The calculation was carried on the website of the sample size calculator: OpenEpi [15]. The minimal sample size required for the study was 385 persons. With an assumed response rate of 75%, a sample size around 469 participants was included.

The sample (n = 469) was distributed over the five urban primary health care centers based on the percentage of the population served by each center relative to the total population served by the five urban primary health care centers selected for the study [16]. For this purpose, the sample selected for each urban primary health care center is presented in Table 1, as organized by the urban primary health care centers. In each urban primary health care center, respondents were chosen at random before giving their approval to participate in the study.

**Table 1:** The sample selected per urban primary health care centers relative to the population served.

UPHCC	Population served in 2019	Percentage (%)	Sample selected per UPHCC

Ihchach	29071	16.11	75
Bouargane	30282	16.78	79
Amsernate	26777	14.84	70
Al Qods	46756	25.92	121
Hay Al Mohammadi	47508	26.33	123
**Total**	180394	100	469

UPHCC: Urban primary health care center, %: Percentage.

### Instrument and Data Collection

A face-to-face questionnaire survey was used for data collection of respondents with the first part including sections reserved for: socio-demographic characteristics (age, sex, marital status, level of education, spoken languages, place of residence, socioeconomic level [family income]), professional occupation (according to the classification of the High Commission for Planning of Kingdom of Morocco), health insurance, body mass index, regular physical exercises, medical history and associated comorbidities (high blood pressure, diabetes, hypercholesterolemia, cardiac disease, history of stroke in respondent or immediate family, and history of stroke in relatives, acquaintances, or neighbors), toxic habits (smoking, alcohol consumption). In addition, a second part includes questions exploring the general knowledge about stroke, its risk factors, as well as the warning signs of a stroke.

Patients were asked to identify risk factors and warning signs. For this survey, the risk factors of stroke were derived from the list established through the INTERSTROKE study [17]. Therefore, high blood pressure, diabetes, smoking, hypercholesterolemia, sedentary lifestyle, obesity, cardiac disease, unhealthy diet, oral contraceptive use, excessive alcohol consumption, previous stroke and family history of stroke were the selected risk factors of stroke.

The warning signs were shown to participants in a list format, and were derived from Schneider et al.’s US survey [18]. These included sudden numbness or weakness in the face, arm or leg; sudden confusion, trouble speaking or understanding others; sudden poor vision in one or both eyes; sudden dizziness, difficulty walking or loss of balance; and sudden headache with no known cause.

Twenty-two questions were used to assess the respondents’ level of knowledge on stroke. The first component focused on generalities about stroke (4 questions), a second related specifically to risk factors for stroke (13 questions), and a third concentrated on warning signs of stroke (5 questions).

One point was awarded for each correct answer given, and zero for any other answer. The sum of all points obtained was converted into a knowledge score of up to 22 points.

Two groups were generated using the K-means clustering method: a group with a high level of knowledge (n = 205 persons) and an average knowledge score of 15, and another group with a low level of knowledge (n = 264 persons) and an average knowledge score of 4.

### Data management and statistical analysis

The qualitative variables were presented as frequency and percentages, with mean ± standard deviation (SD) or median (interquartile range, IQR) for quantitative variables.

The Chi-square test (χ^2^) or Fisher’s exact test, were performed according to their particular application conditions, to look for differences in proportions of categorical variables between two groups (group of respondents with a low level of knowledge on stroke and those with a high level of knowledge on stroke).

Furthermore, univariate and multivariate logistic regression analyses were conducted to identify factors associated with the low level of stroke knowledge in the study population. All independent variables with a p-value <0.25 in the univariate analysis were taken into account in the multivariate logistic regression analysis. P values <0.05 were considered to indicate statistical significance.

Data management and statistical analysis was done using the SPSS for Windows software package (ver. 13.0; SPSS Inc., Chicago, IL, USA).

### Ethics approval and consent to participate

The study has been approved by the ethics committee for biomedical research of the Mohammed V Faculty of Medicine and Pharmacy in Rabat **(N/R: Folder Number 18/20)**, and informed consent was obtained from each subject.

## Results

### Sociodemographic and clinical characteristics of the study sample

A total of 469 participants were surveyed in the study. The population consisted of 190 men (40.5%) and 279 women (59.5%) with an M/F ratio of 0.68. The average age was 38.86 ± 17.01 years with extremes of (18–87) years. The median age was 35 years with an IQR of (23–51).

High blood pressure was reported in 143 persons or 30.5% of the study population, diabetes in 126 or 26.9%, dyslipidemia in 40 or 8.5%, cardiopathy in 38 or 8.1%. A history of stroke was reported in 21 respondents, or 4.5%. A history of stroke was found in immediate family in 129 (27.5%). Two hundred and seventy-six persons (n = 276), or 58.8%, had a history of stroke among relatives, acquaintances, or neighbors (Table 2).

**Table 2:** Level of knowledge about stroke according to sociodemographic and clinical characteristics of the study sample.

Variable	Number (%)	Low level of knowledge n (%)	High level of knowledge n (%)	P value

**Age (years)**				0.0085
18–45	324 (69.1)	169 (36)	155 (33)	
46–65	106 (22.6)	67 (14.3)	39 (8.3)	
≥66 ans	39 (8.3)	28 (6)	11 (2.3)	
**Sex**				0.055†
Male	190 (40.5)	98 (20.9)	92 (19.6)	
Female	279 (59.5)	166 (35.4)	113 (24.1)	
**Marital status**				0.300†
Without a partner^₸^	187 (39.9)	102 (21.7)	85 (18.1)	
In couple (Married)	282 (60.1)	162 (34.5)	120 (25.6)	
**Level of education**				<0.001†
Illiterate	138 (29.4)	93 (19.8)	45 (9.6)	
Primary school	59 (12.6)	44 (9.4)	15 (3.2)	
Secondary school	111 (23.7)	62 (13.2)	49 (10.4)	
University	161 (34.3)	65 (13.9)	96 (20.5)	
**Spoken Languages**				
Dialectal Arabic	287 (61.2)	145 (30.9)	142 (30.3)	0.003†
Amazigh	166 (35.4)	109 (23.2)	57 (12.2)	
Hassaniya	16 (3.4)	10 (2.1)	6 (1.3)	
**Place of residence**				0.003†
Rural	260 (55.4)	133 (28.4)	76 (16.2)	
Urban	209 (44.6)	131 (27.9)	129 (27.5)	
**Socioeconomic level** (Family income)				0.064†
Rich	13 (2.8)	6 (1.3)	7 (1.5)	
Middle class	429 (91.5)	238 (50.7)	191 (40.7)	
Poor	27 (5.8)	20 (4.3)	7 (1.5)	
**Health coverage**				
Health Insurance Obligatory	163 (34.8)	63 (13.4)	100 (21.3)	<0.001†
Medical Assistance Regime (aid)	79 (16.8)	55 (11.7)	24 (5.1)	
Private assurance	26 (5.5)	14 (3)	12 (2.6)	
Others	8 (1.7)	5 (1.1)	3 (0.6)	
None	193 (41.2)	127 (27.1)	66 (14.1)	
**Professional Occupation**				0.047†
Salaried/Employee**^†^**	102 (21.7)	47 (10)	55 (11.7)	
Self-employed worker^∏^	75 (16)	41 (8.7)	34 (7.2)	
Inactive (Unemployed)^∑^	267 (56.9)	160 (34.1)	107 (22.8)	
Retired	25 (5.3)	16 (3.4)	9 (1.9)	
**Obesity or overweight**				
No notion of obesity or overweight	301 (64.2)	159 (33.9)	142 (30.3)	0.027†
With notion of obesity or overweight	168 (35.8)	105 (22.4)	63 (13.4)	
**Diabetes**			0.536†	
Yes	126 (26.9)	71 (15.1)	55 (11.7)	
No	343 (37.1)	193 (41.2)	150 (32)	
**HBP**				0.209†
Yes	143 (30.5)	85 (18.1)	58 (12.4)	
No	326 (69.5)	179 (38.2)	147 (31.3)	
**Hypercholesterolemia**				0.157†
Yes	40 (8.5)	19 (4.1)	21 (4.5)	
No	429 (91.5)	245 (52.2)	184 (39.2)	
**Cardiac disease**				0.048†
Yes	38 (8.1)	16 (3.4)	22 (4.7)	
No	431 (91.9)	248 52.9	183 (39)	
**Smoking**				0.119†
Yes	86 (18.3)	43 (9.2)	43 (9.2)	
No	383 (81.7)	221 (47.1)	162 (34.5)	
**Alcoholism**				0.020†
Yes	28 (6)	10 (2.1)	18 (3.8)	
No	441 (94)	254 (54.2)	187 (39.9)	
**Regular physical exercises**				0.001†
Yes	233 (49.7)	114 (24.3)	119 (25.4)	
No	236 (50.3)	150 (32)	86 (18.3)	
**History of stroke in among the respondent**				<0.001†
Yes	21 (4.5)	3 (0.6)	18 (3.8)	
No	448 (95.5)	261 (55.7)	187 (39.9)	
**History of stroke in the immediate family**				<0.001†
Yes	129 (27.5)	56 (11.9)	73 (15.6)	
No	340 (72.5)	208 (44.3)	132 (28.1)	
**History of stroke among relatives, acquaintances, or neighbors**				<0.001†
Yes	276 (58.8)	120 (25.6)	156 (33.3)	
No	193 (41.2)	144 (30.7)	49 (104)	

%: Percentage, †: The Chi-square test (χ^2^) or Fisher’s exact test, ₸: Includes single, divorced and widowed people, †: According to the High Commission for Planning of Kingdom of Morocco, an employed active person, receiving a salary from a public or private employer, in return for work performed, ∏: According to the High Commission for Planning of Kingdom of Morocco, any employed person working on his or her personal account, running his or her own business or exercising a trade, but who does not employ any employees, ∑: Includes the unemployed, as well as housewives and students. HBP: High blood pressure.

### General knowledge on stroke, risk factors and warning signs of stroke

Concerning study participants’ knowledge regarding generalities on stroke, 78.3% of respondents reported that stroke is a preventable disease, 78.7% indicated that stroke is a curable disease, and 94.5% reported stroke as a pathology requiring urgent managerial actions. Furthermore, approximately 86.6% considered stroke a disabling disease.

Regarding the population’s knowledge of stroke risk factors, high blood pressure was the most reported risk factor for stroke among the respondents at 55.7% followed by depression and stress at 48.8%, previous history of stroke with 37.1%, and smoking at 36.5%.

For warning signs, sudden numbness or weakness in face, arm or leg was mentioned by 37.3%. Similarly, sudden dizziness, difficulty walking or losses of balance, or coordination problems were mentioned by 34.5% of the surveyed population (Table 3).

**Table 3:** Knowledge on stroke.

Variable	Items	Yes Number (%)	No Number (%)

**Stroke is…**			
	A preventable disease	367 (78.3)	102 (21.7)
	A disease requiring an urgent care	443 (94.5)	26 (5.5)
	A curable disease	369 (78.7)	75 (16.0)
	A disabling disease	406 (86.6)	63 (13.4)
**Risk factors for stroke**			
	HBP	261 (55.7)	208 (44.3)
	Diabetes	156 (33.3)	313 (66.7)
	Hypercholesterolemia	125 (26.7)	344 (73.3)
	Cardiac disease	128 (27.3)	341 (72.3)
	Oral contraception	44 (9.4)	425 (90.6)
	Smoking	171 (36.5)	298 (63.5)
	Alcoholism	157 (33.5)	312 (66.5)
	Unhealthy diet	99 (21.1)	370 (78.9)
	Sedentary life style	84 (17.9)	385 (82.1)
	Obesity or overweight	130 (27.7)	339 (72.3)
	Personal history of stroke	174 (37.1)	295 (62.9)
	Family history of stroke	88 (19.0)	380 (81.0)
	Depression and stress	229 (48.8)	240 (51.2)
**Stroke warning signs and symptoms**			
	Sudden numbness or weakness in face, arm or leg	175 (37.3)	294 (62.7)
	Sudden confusion, trouble speaking or understanding others	138 (29.4)	331 (70.6)
	Sudden poor vision in one or both eyes	118 (25.2)	351 (74.8)
	Sudden dizziness, difficulty walking or loss of balance	162 (34.5)	307 (65.6)
	Sudden headache with no known cause	140 (29.9)	329 (70.1)

%: Percentage, HBP: High blood pressure.

### Level of knowledge on stroke among the study population

The average knowledge score was 8.87 ± 5.76. The median knowledge score was 8 (IQR 4–13).

For socio-demographic variables, there is a significant difference between the low level knowledge group and the high level knowledge group according to age (p = 0.0085), level of education (p < 0.001), spoken languages (p = 0.003), place of residence (p = 0.003), health insurance (p < 0.001) and professional occupation (p = 0.047).

Concerning clinical characteristics, a significant difference was found between the low level knowledge group and the high level knowledge group based on: the notion of obesity or overweight (p = 0.027), cardiac disease as associated comorbidity (p = 0.048), regular physical exercise (p = 0.001), history of stroke among the respondents (p < 0.001), history of stroke in immediate family (p < 0.001) and history of stroke among relatives, acquaintances, or neighbors (p < 0.001).

As for toxic habits, a significant difference was reported only between the low level knowledge group and the high level knowledge group in relation to alcoholism (p = 0.020).

Furthermore, there was no significant difference between the group with a low level of knowledge on stroke and the group with a high level of knowledge on stroke based on the presence of some associated comorbidities and toxic habits in the population surveyed (diabetes, high blood pressure, dyslipidemia, smoking, p > 0.05) (Table 2).

### Factors associated with low-level stroke knowledge among the study population

According to the univariate logistic regression analysis: age (18–45 years [OR 2.33; CI_95%_: 1.12–4.85; p = 0.023]), 46–65 years [OR 1.48; CI_95%_: 0.66–3.30; p = 0.^33^]); education level (illiterate [OR 0.32; CI_95%_: 0.20–0.52; p < 0.001], primary school [OR 0.23; CI_95%_: 0.11–0.44; p < 0.001], secondary school [OR 0.53; CI_95%_: 0.32–0.87; p = 0.012]); place of residence (Rural [OR 0.58; CI_95%_: 0.40–0.84; p = 0.004]); obesity or overweight (Yes [OR 1.48; CI_95%_: 1.01–2.18; p = 0.043]); alcoholism (Yes [OR 0.41; CI_95%_: 0.18–0.90; p = 0.028]); regular physical exercises (Yes [OR 1.82; CI_95%_: 1.25–2.63; p = 0.001]); no history of stroke among the respondent (Yes [OR 0.12; CI_95%_: 0.03–0.41; p = 0.001]); history of stroke in immediate family (Yes [OR 2.05; CI_95%_: 1.36–3.09; p = 0.001]); and no history of stroke among relatives, acquaintances, or neighbors (Yes [OR 0.26; CI_95%_: 0.17–0.39; p < 0.01]), were significantly associated with a lower level of knowledge on stroke (Table 4).

**Table 4:** Factors associated with a lower level of knowledge on stroke using univariate and multivariate logistic regression analysis.

Variable	OR (CI 95%)	P value	aOR (CI 95%)	P value

**Age (years)**				
18–45	2.33 (1.12–4.85)	0.023		
46–65	1.48 (0.66–3.30)	0.33		
≥66 ans	1	/		
**Sex**				
Male	1.37 (0.95–2.00)	0.090		
Female	1	/		
**Marital status**				
Without a partner^₸^	1.12 (0.77–1.63)	0.535		
In couple (Married)	1	/		
**Level of education**				
illiterate	0.32 (0.20–0.52)	<0.001	1.92 (1.08–3.44)	0.026
Primary school	0.23 (0.11–0.44)	<0.001	3.43 (1.63–7.21)	0.001
Secondary school	0.53 (0.32–0.87)	0.012	1.37 (0.78–2.40)	0.265
University	1	/	1	/
**Spoken Languages**				
Dialectal Arabic	1.63 (0.57–4.61)	0.35		
Amazigh	0.87 (0.30–2.52)	0.80		
Hassaniya	1	/		
**Place of residence**				
Rural	0.58 (0.40–0.84)	0.004	1.67 (1.07–2.59)	0.023
Urban	1	/	1	/
**Socioeconomic level** (Family income)				
Rich	3.33 (0.83–13.37)	0.089		
Medium class	2.29 (0.95–5.53)	0.065		
Poor	1	/		
**Health coverage**				
Health Insurance Obligatory	2.64 (0.61–11.45)	0.19		
Medical Assistance Regime (aid)	0.72 (0.16–3.29)	0.67		
Private assurance	1.42 (0.28–7.26)	0.66		
Others	0.86 (0.20–3.73)	0.84		
None	1	/		
**Professional Occupation**				
Salaried/Employee^†^	2.08 (0.84–5.14)	0.11		
Self-employed worker^∏^	1.47(0.57–3.75)	0.41		
Inactive (Unemployed)^∑^	1.18 (0.50–2.78)	0.69		
Retired	1	/		
**Obesity or overweight**				
No notion of obesity or overweight	1.48 (1.01–2.18)	0.043		
With notion of obesity or overweight	1	/		
**Diabetes**				
Yes	0.99 (0.66–1.50)	0.98		
No	1	/		
**HBP**				
Yes	0.83 (0.55–1.23)	0.36		
No	1	/		
**Hypercholesterolemia**				
Yes	1.47 (0.76–2.81)	0.243		
No	1	/		
**Cardiac disease**				
Yes	1.86 (0.95–3.64)	0.069		
No	1	/		
**Smoking**				
Yes	1.36 (0.85–2.18)	0.194		
No	1	/		
**Alcoholism**				
Yes	0.41 (0.18–0.90)	0.028		
No	1	/		
**Regular physical exercises**				
Yes	1.82 (1.25–2.63)	0.001		
No	1	/		
**No history of stroke among the respondent**				
Yes	0.12 (0.03–0.41)	0.001	16.41 (4.37–61.59)	<0.001
No	1	/	1	/
**History of stroke in the immediate family**				
Yes	2.05 (1.36–3.09)	0.001		
No	1	/		
**No history of stroke among relatives, acquaintances, or neighbors**				
Yes	0.26 (0.17–0.39)	<0.001	4.42 (2.81–6.96)	<0.001
No	1	/	1	/

CI: Confidence interval, %: Percentage, aOR: Adjusted odds ratio (95% CI), ₸: Includes single, divorced and widowed people, †: According to the High Commission for Planning of Kingdom of Morocco, an employed active person, receiving a salary from a public or private employer, in return for work performed, ∏: According to the High Commission for Planning of Kingdom of Morocco, any employed person working on his or her personal account, running his or her own business or exercising a trade, but who does not employ any employees, ∑: Includes the unemployed, as well as housewives and students. HBP: High blood pressure.

After introducing the following variables: age, sex, education level, place of residence, socioeconomic level, health insurance, professional occupation, obesity or overweight, hypercholesterolemia as associated comorbidity, notion of cardiac disease as associated comorbidity, smoking, alcoholism, regular physical exercise, no history of stroke among the respondent, history of stroke in immediate family and no history of stroke among relatives, acquaintances, or neighbors in the multivariate regression model, the following factors were significantly associated with a lower level of knowledge on stroke: education level (illiterate [Adjusted OR 1.92; CI_95%_: 1.08–3.44; p = 0.026); primary school [Adjusted OR 3.43; CI_95%_: 1.63–7.21; p = 0.001]), place of residence (Rural [Adjusted OR 1.67; CI_95%_: 1.07–2.59; p = 0.023]), no history of stroke among the respondents (Yes [Adjusted OR 16.41; CI_95%:_ 4.37–61.59; p < 0.001]), and no history of stroke among relatives, acquaintances, or neighbors (Yes [Adjusted OR: 4.42; CI_95%_: 2.81–6.96; p < 0.01]) (Table 5).

**Table 5:** Factors associated with a lower level of knowledge on stroke using multivariate logistic regression analysis.

Variable	aOR (CI 95%)	P value

**Level of education**		
Illiterate	1.92 (1.08–3.44)	0.026
Primary school	3.43 (1.63–7.21)	0.001
**Place of residence**		
Rural	1.67 (1.07–2.59)	0.023
**No history of stroke among the respondent**		
Yes	16.41 (4.37–61.59)	<0.001
**No history of stroke among relatives, acquaintances, or neighbors**		
Yes	4.42 (2.81–6.96)	<0.001

aOR: Adjusted odds ratio (95% CI), CI: Confidence interval, %: Percentage.

## Discussion

In this study, more than three-quarters of the population were aware of the preventable and urgent nature of the stroke. These results are similar to those found in previous studies [19,20]. Also, the majority of respondents mentioned that stroke is a disabling disease, which is consistent with the results found in a study of Arab-Muslim Israelis which highlighted that stroke is always associated with physical burden, disability, and dependence [11].

As for respondents’ knowledge of risk factors for stroke, this study has found that high blood pressure, depression and stress were the most well-known risk factors with a percentage near 50%. This is similar to the results of a wide range of studies conducted in several countries [11,12,13,19,21,22,23,24,25]. A remarkable lack of knowledge of the population regarding the risk factors for stroke, and especially the most well-known and classic ones, have been detected in our context. By this logic, two-thirds did not recognize diabetes or hypercholesterolemia as risk factors for stroke and almost half of the population did not recognize high blood pressure as a risk factor for stroke.

These results could be explained by the limited and insufficient access of the Moroccan population to services related to the diagnosis, treatment, and control of non-communicable diseases provided in primary health care centers. Additionally, a significant segment of the population uses unconventional and traditional medicine, which would limit their chances to be educated about and raise awareness of risk factors [26].

Moreover, the majority of the surveyed participants showed an unsatisfying level of awareness regarding warning signs of a stroke. This result could be explained in macroscopic context, by the lack of mass education and awareness campaigns for the benefit of the general public. A few campaigns are occasionally organized on World Stroke Day, usually in cities where a university hospital is based, in which the acronym FAST (F: Face, A: Arm, S: Speech, T: Time) is adapted in dialectal Arabic language for use in the awareness campaign educational materials. Additionally, this low level of warning sign recognition could be linked at the microscopic level to the lack of individualized awareness sessions at the first signs suggestive of stroke, which would benefit the Moroccan population and, more specifically, people at cardiovascular risk in the context of medical consultations.

This lack of knowledge of the warning signs of stroke potentially impacts on the early use of specialized hospital centers for possible management of stroke patients. This finding was missed in a recent Moroccan systematic review study [6]. To address this concern, the High Authority of Health in France recommended that the treating physician inform patients at risk (vascular history, high blood pressure, diabetes, arteriopathy of the lower limbs, and so on), as well as their entourage, about the main signs of stroke to contribute to rapid access to neurovascular units [27].

Overall, this study revealed there is clearly a poor level of knowledge in the population surveyed about stroke. This is identical to the findings in several countries around the world [10,11,12,28]. However, other investigations have shown a good level of knowledge about this disabling disease [19,29].

In this regard, the variability in the level of knowledge of the population regarding stroke in studies is the expression of a phenomenon whose determinants are multiple. The present study revealed in the multivariate logistic regression analysis that illiteracy, primary school, rural residential, no history of stroke among the respondent and no history of stroke among relatives, acquaintances, or neighbors were independently associated with a lower level of knowledge about stroke.

The low level of education has been associated in the Moroccan context with a poor level of knowledge, the Souss Massa region illiteracy rate (33.1%) being slightly higher than the 2018 national rate (32.2%) reported by the High Commission for Planning of Morocco [30]. This is consistent with the results of a range of studies in which low education level has been the factor most associated with a low level of knowledge in the population surveyed about stroke [19,31,32,33]. Similarly, other investigations have confirmed an association between a higher level of education and a good state of knowledge [12,34,35,36].

As for place of residence and its relationship to the level of awareness of the surveyed population, this could be explained by access to healthy lifestyle advice for the population living in urban areas, unlike that of rural areas, confirmed recently by the results of the national survey on common risk factors for non-communicable diseases [26]. On the other hand, there was a study conducted in Mexico, which suggested that due to the increased frequency of awareness and information campaigns in rural areas and due to the consolidated “physician-patient” relationship in rural primary health care centers that more preventive education on common cardiovascular disorders, such as stroke, may be found [37].

Moreover, as a result of a first stroke, the risk of a new incident increases considerably. These recurrent strokes account for 25–30% of all strokes as a result of the failure of secondary prevention, and they are probably more disabling and more likely to be fatal than initial strokes [38,39]. Since the state of knowledge among stroke survivors is of crucial importance in the secondary prevention of recurrent strokes, it has been demonstrated, in present investigation, that a personal history of stroke is a protective factor against a low level of knowledge. This result is similar to that found in several investigations [36,40,41,42], while other studies have shown the persistence of a low level of knowledge in patients surviving after a stroke [43,44,45,46]. Similarly, a case-control study has found that the level of knowledge in patients after a stroke or transient ischemic accident was low compared to randomly select healthy individuals [47]. This could be explained by the individualized information and awareness sessions conducted in the hospital setting by health professionals involved in the management of stroke patients, which generates an accumulation of knowledge related to the disease throughout the care pathways. Presumably, it could be the consequence of anxiety about the risk of having another stroke, which develops a curiosity in patients to know additional details concerning the disease, especially those for whom the unexpected occurrence of the stroke induces an anxious anticipatory state [48].

The no history of stroke among relatives, acquaintances, or neighbors is found to be a risk factor for a low level of knowledge. This result is probably due to the consolidated interpersonal and social relations with patients in the Moroccan community during visits.

Bolstering this result is a French study which has demonstrated the importance of interpersonal contact in the dissemination of medical information and, more specifically, information about stroke [49]. In another study, a parent was shown to be the primary source of knowledge. To this end, the education of a single person within a family could play a crucial role in raising public awareness of stroke [10].

This study has several limitations. The location of the study constitutes the first constraint, which has focused exclusively on people attending urban primary health care centers despite the recruitment of rural residents with a percentage close to 50%. Another limitation is related to the cross-sectional nature of the study, which reflects only the current level of knowledge of the population surveyed and does not take into account changes over time. Additionally, the adoption of questions about risk factors and warning signs in the list format may result in an overestimation of the current knowledge of the surveyed population.

## Conclusion

This study showed important lack of knowledge about risk factors and warning signs of stroke in this sample of the Moroccan population. There is a need to adopt the community-based approach focused on the delegation of education and awareness tasks to experts’ patients, stroke survivors or patients’ caregivers, such as community health workers (relays). This is to implement proximity prevention programs characterized by flexibility at the temporospatial level to meet the specificities and real needs of communities in terms of education and awareness, to replace the human and logistical constraints associated with the implementation of education and awareness campaigns of the general Moroccan public.

Such a poor disease knowledge is strongly correlated to the low educational level. Thus, this indicator calls for further development of sociological studies in order to strengthen the therapeutic protocols taking into account the social status of patients, their cultural context, their ability to verbalize, their perception of the disease, and of the medical language.

## Data Accessibility Statement

All data generated or analyzed during this study are included in this published article.
